# A Review of “My Life in Science: The Story of Biotinidase Deficiency” by Dr. Barry Wolf

**DOI:** 10.3390/ijns11040111

**Published:** 2025-12-04

**Authors:** Harvey L. Levy

**Affiliations:** Division of Genetics and Genomics, Department of Medicine, Boston Children’s Hospital, Boston, MA 02115, USA; harvey.levy@childrens.harvard.edu

This book by Dr. Wolf, who not only discovered **Biotinidase deficiency** but also developed and published everything we know about the disease, constitutes a very interesting and totally honest memoir, a virtual syllabus on how one becomes a physician scientist and then uses his scientific as well as medical knowledge to discover and fully characterize a single disease. He not only describes his life in astonishing detail, but also describes biotinidase deficiency in both scientific and clinical terms. 

In the first part of the book, Dr. Wolf recounts his early life, from birth through childhood and into college and medical school. Notably, he was the only boy in a very close extended family in the Chicago area who was doted on by his relatives and was especially close to his accomplished father who, although not educated beyond high school, had the mind of a scientist and fostered science in his totally receptive son. The reader will clearly understand how Dr. Wolf became a highly successful scientist. In the second part of the book, Dr. Wolf recounts both the events in his post-doctoral life that led to his discovery of biotinidase deficiency, as well as the biochemical, genetic and clinical details of the discovery, all of which he and his laboratory elucidated. There is probably no other example in the annals of metabolic diseases of a single person not only discovering a disorder but also discovering every basic and clinical aspect of it, including its clinical features, its treatment, its response to treatment, its inheritance, its basic molecular genetic features, its variants, and its newborn screening.

Even though newborn screening takes up a relatively short part of the book, toward the middle and end, readers of *IJNS* are perhaps most likely to find his discovery and advocacy of newborn screening for biotinidase deficiency the most interesting aspect—and, in many ways, it is. It is key not only to understanding the disorder as an important medical entity, because it has led to the prevention of disability in children throughout the world, but is also the aspect of his work that has brought Dr. Wolf the most attention and led to the many accolades and awards he has received. One might say that had he not discovered and developed newborn screening for biotinidase deficiency, the disorder would have been considered an interesting addition to the list of rare inborn errors of metabolism and that biotin therapy might reverse some of the clinical features and prevent further damage but, in the absence of a family history, not be preventive in populations. In this it would have mimicked PKU, in which dietary therapy could also not prevent damage in those without a family history until the development of newborn screening. 

I recommend *My Life in Science* to everyone directly or even indirectly involved in newborn screening, as well as those in all areas of biochemical genetics. It is not only an interesting and very informative read about the history of an important metabolic disorder, but is also very informative about the making of a medical scientist. It has general application that will be enjoyed by many.

It can be purchased on Amazon.

